# Special Issue “Sports Medicine and Public Health”

**DOI:** 10.3390/jfmk11020156

**Published:** 2026-04-16

**Authors:** Giovanni Fiorilli, Andrea Buonsenso

**Affiliations:** 1 Department of Medicine and Health Sciences, University of Molise, 86100 Campobasso, Italy; 2 Department of Theoretical and Applied Sciences, eCampus University, 22060 Novedrate, Italy; andrea.buonsenso@uniecampus.it

## 1. Introduction

In recent years, physical activity has increasingly been recognised as an integral component of public health, contributing substantially to the prevention, management, and treatment of major non-communicable diseases. Within this framework, the concept of “Exercise as Medicine” has emerged as a well-established paradigm, supported by a growing body of evidence from both clinical research and large-scale implementation studies [1,2,3]. This Special Issue, “Sport Medicine and Public Health”, presents twenty-one original and review articles that collectively integrate empirical evidence with applied perspectives. By bridging experimental research and practical implementation, it provides a comprehensive examination of the role of physical activity in health promotion and disease management. The contributions are organised into six principal thematic domains: occupational health and safety, physical activity and exercise, sports medicine and injury, chronic disease and metabolic health, obesity and paediatric health, and sleep and mental health.

## 2. Health and Occupational Safety

The initial thematic focus was on the physiological stressors and injury risks that are characteristic of elite sports and high-risk professional environments. In this context, Wohlgemuth et al. provided an extensive review of strategies to bolster the health of firefighters during active-duty shifts, complemented by Simonson et al., who explored the distinct correlations between dietary habits and cardiovascular health risks among American career firefighters. Transitioning to the high-performance maritime sector, Linvill et al. conducted a retrospective cohort study to analyse the incidence of injury and illness among male and female sailors in the professional sailing circuit. Together, these studies emphasise that high-stakes occupations require highly specialised and tailored health protocols to maintain operational readiness and long-term wellness.

## 3. Physical Activity and Exercise

As the most substantial cluster within this Special Issue, this section investigates the mechanics of physiological responses, novel training protocols, and the deployment of advanced monitoring technologies. Addressing contemporary challenges, Centorbi et al. examined how structured physical activity can mitigate the lingering symptoms of Long COVID in adults over 40 years of age. Optimisation of performance remains a key sub-theme: Bordoli et al. explored the impact of oral lactate supplementation on acid–base equilibrium during high-intensity cycling, while Lama et al. sought to define the specific cycling cadence thresholds necessary to improve subsequent walking velocity. To ensure that these assessments are as precise as possible, Schelling et al. discussed the integration of decision support systems to refine movement proficiency evaluations.

The interplay between clinical intervention and lifestyle is further highlighted by De Brito Fontana et al., who investigated the combined effects of exercise and prebiotic fibre on diet-related morbidities. Similarly, Papale et al. analysed the efficacy of multidimensional activity programmes on glycaemic stability in patients with type 1 diabetes. Community-level impact was demonstrated by Atti et al., whose work showed that supervised team sports within municipal health settings can effectively reduce systolic blood pressure in individuals with hypertensive chronic obstructive pulmonary disease and Type 2 diabetes. Furthermore, Saavedra et al. introduced a replicable training load monitoring system for those with sleep-disordered breathing. Finally, Fiorilli et al. underscore the cognitive dimension of movement, whose randomised trial showed that active breaks in the classroom significantly boost processing speed and mathematical performance in primary school students.

## 4. Sports Medicine and Injury

This pillar investigates the physiological impact of high-intensity combat sports and the evolving landscape of concussion management and rehabilitation in such sports. Niewczas et al. characterised the acute haematological responses associated with kickboxing competition. Concerning neurological function, Walshe et al. appraised the diagnostic and clinical relevance of ocular assessments in the management of sport-related concussions, while Bolduc et al. investigated the prolonged disruption of sympathovagal balance in athletes undertaking complex dual-task paradigms following head injury. In the field of physical recovery, Rodrigo-Mallorca et al. assessed the benefits of superimposed blood flow restriction during isokinetic knee extension, suggesting a path toward more efficient post-injury rehabilitation.

## 5. Chronic Disease and Metabolic Health

This section focuses on lifestyle modifications and management of oxidative stress in clinical populations. Núñez-Cortés et al. presented a strong case for physical activity as the primary driver of lifestyle changes in the management of chronic musculoskeletal pain. At the cellular level, Moldovan et al. utilised animal models to provide ultrastructural analysis of oxidative stress across multiple organ systems following sprint exercises. Bridging the gap to professional life, Maisto et al. documented the results of a 24-week workplace physical activity intervention, noting significant improvements in metabolic health markers and physical fitness among academic staff.

## 6. Obesity and Childhood Health

The preventive efficacy of interventions implemented during early life constitutes a recurring focus in public health discourse. Porri et al. identified the development of functional motor competence as a pivotal strategy in the prevention of childhood obesity. Their evidence supports the premise that early movement proficiency is integral to the maintenance of long-term metabolic homeostasis, serving as a protective factor against the onset of obesity-related pathologies in later stages of life.

## 7. Sleep and Mental Health

The final thematic pillar examined the relationship between physical rehabilitation and neurophysiological integrity. Carolina et al. evaluated a progressive kinesiotaping protocol for women with chronic lower back pain, utilising electroencephalography to monitor the brain response to treatment. This study effectively bridges the gap between musculoskeletal therapy and mental health, illustrating that the path to physical recovery and neurological stability is often interlinked.

## 8. Conclusions and Future Perspectives

The twenty-one contributions within this Special Issue provide clear evidence that sports medicine has evolved into a cornerstone of modern public health. Collectively, these studies point to a clear trajectory for future practice. However, the translation of this evidence into scalable and sustainable public health strategies capable of reaching diverse communities remains a primary challenge. The emergence of digital technologies and wearable devices has expanded the capacity for individualised monitoring, positioning exercise as a potentially high precision therapeutic modality. However, the translation of these innovations into practice requires coherent integration into existing healthcare systems and regulatory frameworks. Therefore, physical activity should be treated with the same level of rigour and institutional support as established pharmacological interventions.

Taken together, the contributions in this Special Issue reinforce the conceptualisation of sports medicine and public health as fundamentally interconnected domains within the broader framework of human health. By merging performance science with population health perspectives, we hope to establish physical activity as a central, non-negotiable element of modern prevention, clinical treatment, and long-term well-being.
